# The prevalence of schizophrenia and other psychotic disorders among homeless people: a systematic review and meta-analysis

**DOI:** 10.1186/s12888-019-2361-7

**Published:** 2019-11-27

**Authors:** Getinet Ayano, Getachew Tesfaw, Shegaye Shumet

**Affiliations:** 1 Research and Training Department, Amanuel Mental Specialized Hospital, Addis Ababa, Ethiopia; 2 Department of Psychiatry, College of Medicine and Health Sciences, University of Gondar, Gondar, Ethiopia

**Keywords:** Homeless, Schizophrenia, Psychotic disorder, Systematic review, Meta-analysis

## Abstract

**Background:**

Schizophrenia and other psychotic disorders constitute a huge global burden of disease and they are major contributors to disability as well as premature mortality among homeless people. This systematic review and meta-analysis aimed to estimate the pooled prevalence of schizophrenia and other psychotic disorders among homeless people.

**Methods:**

PubMed, Embase, and Scopus were searched to identify pertinent studies. We used a fixed- or random-effect meta-analysis to pool data from the included studies depending on the anticipated heterogeneity. A predesigned search strategy, as well as inclusion and exclusion criteria, were used. We also performed subgroup and sensitivity analysis and Cochran’s Q- and the I^2^ test was employed to compute heterogeneity. Egger’s test and visual inspection of the symmetry in funnel plots were used to assess publication bias.

**Results:**

Thirty-one studies involving 51,925 homeless people were included in the final analysis. The meta-analysis showed a remarkably higher prevalence of psychosis [21.21% (95% CI:13.73, 31.29), I^2^ = 99.43%], schizophrenia [10.29% (95%, CI: 6.44, 16.02), I^2^ = 98.76%], schizophreniform disorder [2.48% (95% CI: 6.16, 28.11), I^2^ = 88.84%] schizoaffective disorder [3.53% (95% CI: 1.33, 9.05), I^2^ = 31.63%,] as well as psychotic disorders not otherwise specified [9% (95% CI: 6.92, 11.62), I^2^ = 33.38%] among homeless people. The prevalence estimate of psychosis was higher in developing (29.16%) as compared to developed (18.80%) countries. Similarly, the prevalence of schizophrenia was highest in developing (22.15%) than developed (8.83%) countries.

**Conclusion:**

This systematic review and meta-analysis revealed that schizophrenia and other psychotic disorders are highly prevalent among homeless people, indicating an urgent need for studies to help develop better mechanisms of prevention, detection as well as treatment of those disorders among homeless people.

## Background

Globally, as many as 100 million people were categorized as homeless, and over 1.6 billion individuals lacked adequate housing [1]. A wide range of multiple and interrelated factors contributes to homelessness such as domestic violence, social isolation, unemployment, addictions, mental illness, sexual assault, poverty, a critical shortage of affordable housing, financial difficulties, family breakdown, and adverse childhood experiences [2–6].

As compared to the general population, homeless people suffer from a greater prevalence of psychiatric disorders including schizophrenia and other psychotic disorders [7, 8]. The reported magnitude of mental disorders among homeless people ranged from 25 to 50% across the studies [9–11]. This prevalence rate rises to about 92% among those who are street homeless [7]. The presence of mental disorders among homeless people is linked with a higher risk of disability and mortality from suicide and general medical [12–14], as well as alcohol and drug-related causes [15].

Epidemiologic evidence from several studies has shown that schizophrenia and other psychotic disorders are among the most common psychiatric disorders among homeless people [7, 8, 16] and they are associated with a greater risk of comorbid physical disease, substance use, and disability as well as mortality from different causes [12–14, 16–18]. The reported prevalence estimates of schizophrenia among the homeless people range from 2.29 to 60% depending on the studies [7, 8, 16–22]. The impacts of psychotic disorders among homeless people could be severe and extensive, it negatively impacts the homeless person suffering from it, the family, relatives and other population [12, 15, 23, 24].

A better understanding of the epidemiology of schizophrenia and other psychotic disorders among the homeless people could assist the development of better policies and intervention strategies as well as best intervention mechanisms to address the impacts associated with these disorders. Although there are previous systematic reviews on overall mental disorders and psychosis among homeless people, to our knowledge, this is the first systematic review and meta-analysis aimed to provide the pooled prevalence estimates of schizophrenia and other psychotic disorders among the homeless. we hypothesized that the prevalence of schizophrenia and other psychotic disorders are notably high among homeless people. Therefore, the purpose of this study was to evaluate the results of available epidemiologic studies in order to provide a high level of evidence on the global prevalence of schizophrenia and other psychotic disorders among homeless and forward implications of the findings for the future clinical practice as well as research.

## Methods

### Research design and method

We conduct this systematic review and meta-analysis in adherence to the PRISMA (Preferred Reporting Items for Systematic Reviews and Meta-Analyses) guidelines [25]. A predesigned protocol was utilized for searching, data abstraction, inclusion-exclusion criteria, quality evaluation, as well as data synthesis and analysis.

### Data source and selection process

The electronic search was performed in the electronic databases (PubMed, Embase, and Scopus). We conducted our search in PubMed using the following terms: (schizophrenia OR schizophreniform OR schizoaffective OR psychotic disorder OR psychiatric disorder OR mental disorder)) AND (prevalence OR magnitude)) AND (homeless). Embase and Scopus were searched using specific-subjects headings suitable for the databases. We also performed a manual search to supplement our electronic database search.

### Inclusion and exclusion criteria

Studies satisfying the following criteria were included in this systematic review and meta-analysis: (1) Conducted using observation study in design (cross-sectional, case-control or cohort studies). (2) The study population was homeless people (conducted among homeless people). (3) Estimated the prevalence of schizophrenia and other psychotic disorders or data to calculate the prevalence. Thus, commentaries, conference presentations, reviews, book reviews, and studies conducted in the non-homeless population were excluded.

### Methods for data extraction and quality assessment

Two authors (SS and GT), independently performed data abstraction from the included studies., We used predefined data extraction form to extract pertinent data from the eligible studies as advised by PRISMA guidelines [25]. The following data were extracted from all each study: author(s) name, tools used, country of the study, sample size, year of publication, and a number of positive cases, and magnitude of schizophrenia and other psychotic disorders. The quality of the studies was evaluated a modified version of the Newcastle-Ottawa Scale (NOS) [26]. The NOS grading standard is based on three main domains such as group selection (contains 4 items), comparability across the groups (one item), and assessment of exposure and outcome (4 items). The total scores including all items range from zero to nine, the highest score indicating greater quality study. The NOS has excellent test-retest reliability and inter-rater reliability ranging from poor to fair depending on the components [27].

### Definitions of key terms and concepts

#### Developed and developing countries

In this review, those countries with both longstanding established market economy as well as longstanding strong traditions in the research were considered as developed countries [28]. Thus, the USA, Canada, German, Spain, France, Scotland, the UK, Japan, and Australia were those countries, which satisfied the criteria for developed countries. Other countries such as China, Ethiopia, Ireland, and Serbia were considered as developing countries.

#### Diagnostic definitions

In the Diagnostic Statistical Manual of Mental disorders fifth, Edition, text revised (DSM-5), disorders having psychosis (including delusion, hallucinations, disorganized speech, and behaviors) as their primary manifestations are considered as schizophrenia and other psychotic disorders. According to this definition schizophrenia, schizoaffective, schizophreniform, delusional, brief psychotic disorders, and psychotic disorders due to another medical condition/substance were included under schizophrenia and other psychotic disorders [29]. We also included studies that used the previous diagnostic manual criteria such as the Diagnostic Statistical Manual of Mental disorders fourth, Edition, text revised (DSM IV) criteria [30]. Those studies that reported psychosis among homeless people without labeling the psychotic presentations under the above specific categories are considered separately as having psychosis (overall psychosis).

#### Homelessness

Globally, there is no consistent definition of homelessness. Some countries define homelessness as the absence of access to safe shelter and some countries define broadly including those people who are sleeping public places or designed shelter, people living in marginal accommodations (marginal housing) and rooflessness [31, 32]. In this systematic review and meta-analysis, homelessness represents the broadest definition (which is beyond rooflessness) because we have included studies, which defined homelessness in various ways.

#### Data synthesis and analysis

All statistical analysis was conducted by using comprehensive meta-analysis software version 3 [33]. Evaluation of the heterogeneity among the studies was performed using Cochran’s Q Test and the I^2^statistics [34]. The explained variance due to heterogeneity between studies was evaluated by I^2^ statistics [34] and the I^2^ indexes 25, 50 and 75% denoted low, medium and high, respectively [35]. For those studies, which were identified as heterogeneous, we conducted the analysis based on a random effect model, which is a conservative model than a fixed-effect model and is the advisable model to pool the results in the cases of heterogeneity [36]. Sensitivity and subgroup analyses were conducted based on tools used to measure schizophrenia and other psychotic disorders, the country of the study, and the quality of the studies. However, we used a fixed-effect meta-analysis for those outcomes where there is no significant heterogeneity between the studies. We used 2-tailed t-Test to evaluate whether the observed difference is statistically significant or occurred by chance. Publication bias was evaluated using a funnel plot and Egger’s regression tests. All the reported probabilities were two-sided and *P*-value < 0.05 considered statistically significant.

We also conducted a meta-regression to quantify the impacts of the year of the study (studies conducted before 2010 and 2010 and above), country status (developing and developed), and, and the instrument used to measure the disorders (screening and diagnostic) on the observed heterogeneity across the studies.

## Results

### Identification of studies

**Fig. 1:**
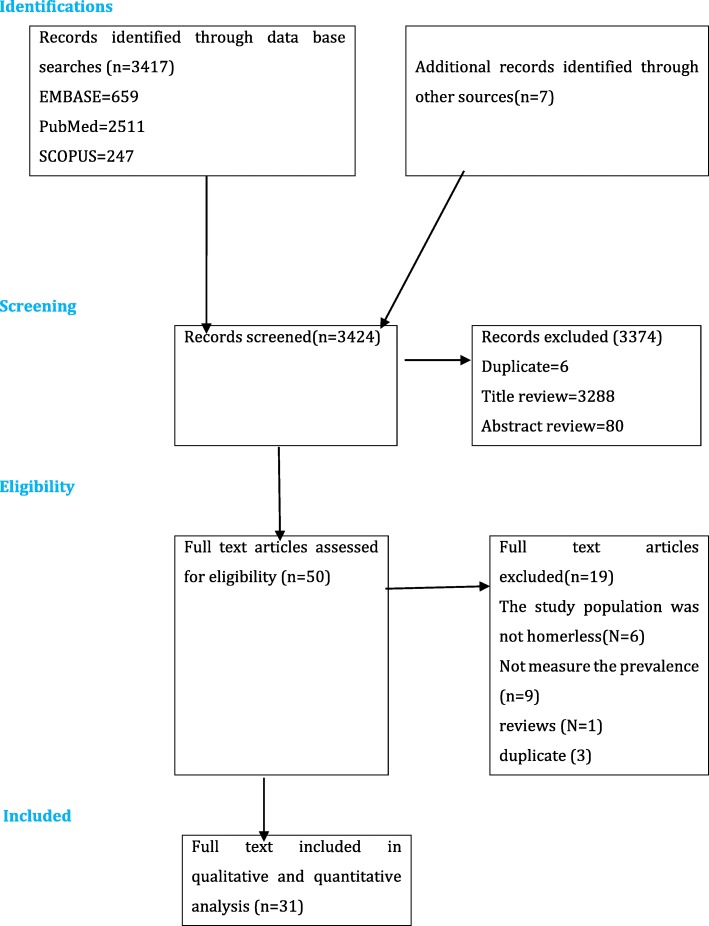
PRISMA flowchart of review search. This figure shows the process of searching of pertinent studies form the three databases including identification, selection, eligibility and inclusion of the studies depending on the predefined criteria

Figures 1 illustrates the PRISMA flow chart of the search process. Three thousand four hundred seventeen studies were potentially detected by our electronic search engine and strategies. Additionally, we detected 7 studies by our manual search making the total studies 3424. Of these, 3294 were excluded during the assessment of duplicate and titles, as they did not meet the eligibility criteria. Our assessment of abstract in strict accordance with the inclusion and exclusion criteria resulted in the exclusion of a further 80 articles. Thus, a full-text of 50 articles were retained for further assessment and 19 of these were excluded. (Additional files 1 and 2).

### Characteristics of included studies

In this systematic review and meta-analysis, a total of 31 studies [7, 20–22, 37–63] were included in the final analysis. The studies were conducted in 13 countries representing 51,925 participants. Table 1 illustrates the characteristics of the articles included in this study. Of the 31 studies, 12 were conducted in the USA [37–40, 42–44, 47, 49, 55, 58, 60], four were conducted in Canada [45, 48, 57, 59], three conducted in German [20, 21, 41], two conducted in Ethiopia [7, 51], two conducted in France [22, 62], two conducted in Scotland) [56, 61], one conducted in China [63], one conducted in Spain [38], one conducted in the UK [50], one conducted in Japan [52], one conducted in Ireland [53], one conducted in Serbia [46], and one conducted in Australia [54]. The studies were published between 1984, and 2017, with the sample size ranging between 33 participants in the German and 29,143 participants in Canada.

**Table 1:** Distribution of studies on psychosis in people living with homeless included in qualitative and quantitative analysis based on year, study design, sample size, instrument, country, response rate, study population and prevalence

Author (year) (reference number)	Sample size	Tool	Country	Outcome (magnitude of psychosis)
(Susser et al., 1989) [37]	223	DSM	USA	8%(*n* = 18) schizophrenia
(Munoz et al., 1998) [38]	Madrid = 262 1563 = USA	CIDI	Spain & USA	Madrid; 2.4%(*n* = 6) Los Angeles 5.5%(*n* = 86) Schizophrenia
(Bassuk et al., 1986) [39]	80	DSM	USA	3%(*n* = 2) Schizophrenia
(Koegel et al., 1988) [40]	328	DSM	USA	13.7%(*n* = 45) Schizophrenia / schizophreniform 13.1%(*n* = 44) schizophrenia 0.6%(*n* = 2) schizophreniform
(Fichter and Quadflieg, 2001) [20]	265	DSMIII	Germany	9.8%(*n* = 26) psychosis 4.4%(*n* = 12) schizophrenia
(Längle et al., 2005) [41]	99	ICD and DSM	Germany	11%(*n* = 7) Psychosis disorders
(Connolly et al., 2008) [42]	60	DSM-IV	USA	20%(*n* = 12) psychosis 10%(*n* = 6) schizophrenia 5%(*n* = 3) schizoaffective 5%(*n* = 3) psychosis NOS
(Ghose et al., 2013) [43]	2898	ICD	USA	3.5%(*n* = 98) Schizophrenia
(Tsai et al., 2014) [44]	29,143	DSM	USA	9.3%(*n* = 2707) Psychosis
(Noe et al., 2016) [45]	497	DSM	Canada	*N* = 263 (53%) Psychosis
(Sarajlija et al., 2014) [46]	104	ICD	Serbia	35.6% psychosis (*n* = 37) *N* = 11 (10.6%) Schizophrenia
(Ayano et al., 2017) [7]	456	SCID)	Ethiopia	Schizophrenia *N* = 274 (60.09%) Psychotic disorders NOS *N* = 45 (9.87%)
(Schinka et al., 2012) [47]	10,111	DSM	USA	Schizophrenia = 432 (4.27%) Other psychosis = 420 (4.15%)
(Topolovec-Vranic et al., 2017) [48]	2088	MINI	Canada	35%(*N* = 731) Psychosis
(Fletcher and Reback, 2017) [49]	131	SCID	USA	22%%(*n* = 29) Psychosis
(Cuvee.Adams et al., 1996) [50]	64	DSM	UK	42%(*n* = 27) Schizophrenia
(Fekadu1 et al., 2014) [51]	217	CANSAS	Ethiopia	41%(*n* = 89) Psychosis Schizophrenia (*n* = 79)
(Okamura et al., 2014) [52]	346	WHO-Five Well-Being Index (WHO-5-J)	Japan	8.1%(*n* = 28) Schizophrenia
(Prinsloo et al., 2012) [53]	38	DSM	Ireland	5.3%(*n* = 2) Schizophrenia and Other Psychotic Disorders
(Kovess and Lazarus, 1999) [22]	715	CIDI	France	5.8%(*n* = 42) schizophrenic/delusional disorders (certain)
(Larney et al., 2009) [54]	105	Lifetime (report)	Australia	37.1%(*n* = 39) schizophrenia or other psychotic disorder.
(Fischer et al., 1986) [55]	51	DSM-III	USA	2%(*n* = 1) schizophrenia
(Greifenhagen et.al. 1997) [21]	32	DSM-III	Germen	Schizophrenic disorders *n* = 11 (34%) Schizophrenia *n* = 8 (25%) Schizophreniform disorder *n* = 3((%)
(Newton et al., 9194) [56]	65	DSM	Scotland	3%(*n* = 2) schizophrenia
(Bacciardi et al., 2017) [57]	416	MINI	Canada	26%(*n* = 94) Schizophrenia
(Goldstein et al., 2012) [58]	3595	DSM $ ICD	USA	17.6%(*n* = 631) schizophrenia
(Freeman et al., 1979) [59]	250	Present State Examination (PSE)	Canada	4.4%(*n* = 11) schizophrenia
(Bassuk et al., 1984) [60]	78	DSM	USA	29.5%(*n* = 23) Schizophrenia
(Geddes et al., 1994) [61]	79 in 1966 136 in 1992	DSM	Scotland	25%(*n* = 20) in 1966 9%(*n* = 12) in 1992 Schizophrenia
Cougnard et.al. 2006 [62]	104	ICD	France	32.7% (*n* = 34) psychosis
Yim et.al 2015 [63]	79	SCID	China	Psychotic disorder 10.1% (*n* = 8) Schizophrenia (*n* = 3) Schizoaffective (*n* = 1) Psychotic NOS (*n* = 4)

Schizophrenia and other psychotic disorders among homeless people were predominantly measured using the DSM. The DSM was used in 18 studies, MINI in 4 studies, CIDI in 3 studies, ICD in 3 studies and the others (CANSAS, PSE, WHO-5-Jand self-report) each used in one study.

Regarding the categories of psychotic disorders reported, 18 studies assessed overall psychotic disorder, 23 assessed schizophrenia, 2 assessed schizophreniform, two assessed schizoaffective disorder, 4 assessed psychotic disorder NOS, and one assessed brief psychotic disorder.

### Quality of included studies

We assessed the quality of the studies using NOS with modification. From the total, 18 studies were found to be good quality (NOS score 7 and above), and 13 studies were fair quality (NOS score between 2 and 6 inclusive) (Additional file 3).

### The results of a pooled meta-analysis

#### The prevalence of the overall psychotic disorder in homeless people

**Fig. 2:**
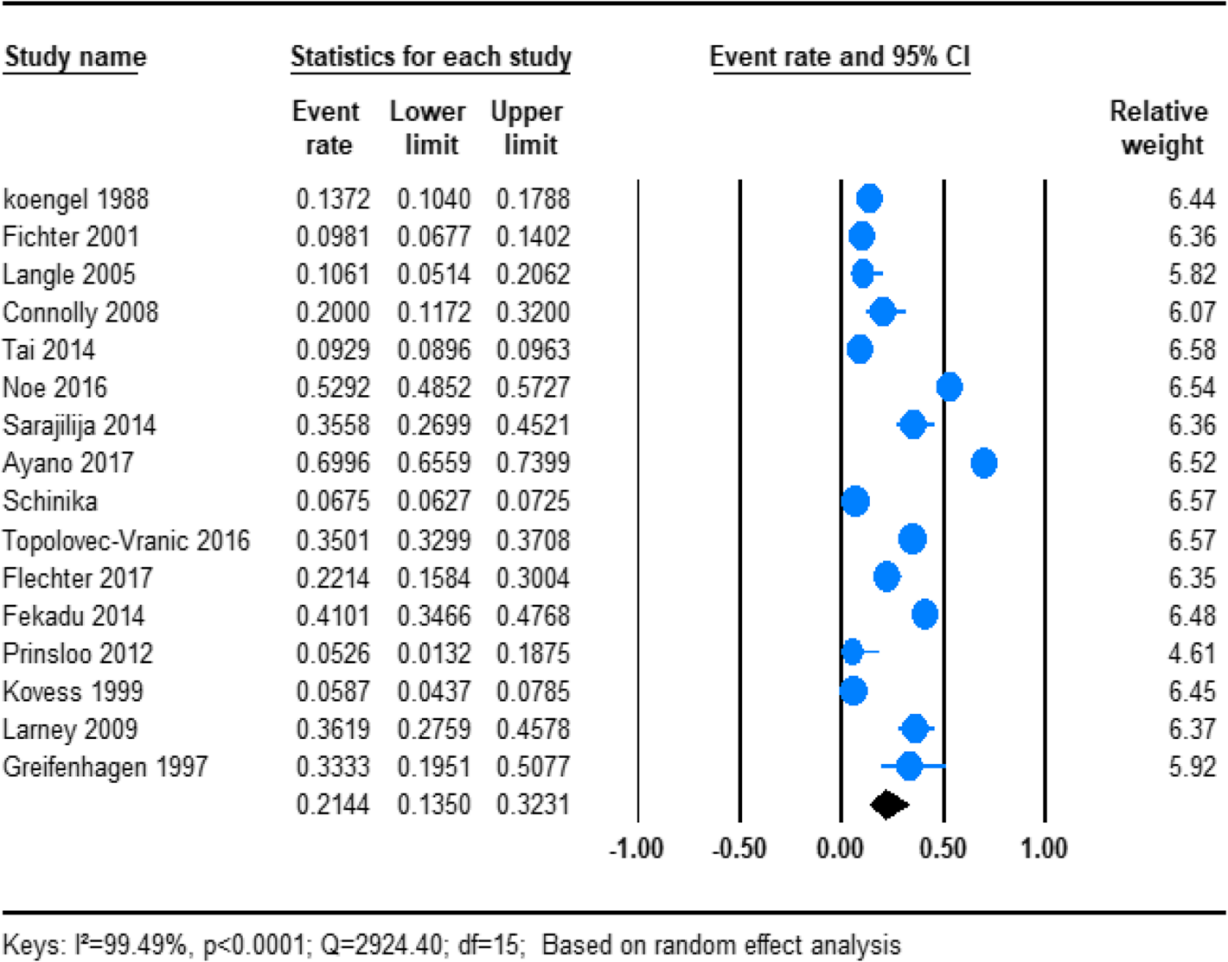
Forest plot of the prevalence of psychosis among homeless people. The figure shows the results of the meta-analysis of the studies on psychosis on homeless people suing random effect model

From the total, 18 of the studies reported the prevalence of the overall psychotic disorder in homeless people (Table 1). Based on the results of the random-effects method, the pooled prevalence estimates of psychotic disorder in the homeless people was 21.21% (95% CI; 13.73–31.29). We found significant heterogeneity for this analysis (*I*^2^ = 99.43%; *p* < 0.001) (Fig. 2).

#### The prevalence of schizophrenia in homeless people

**Fig. 3:**
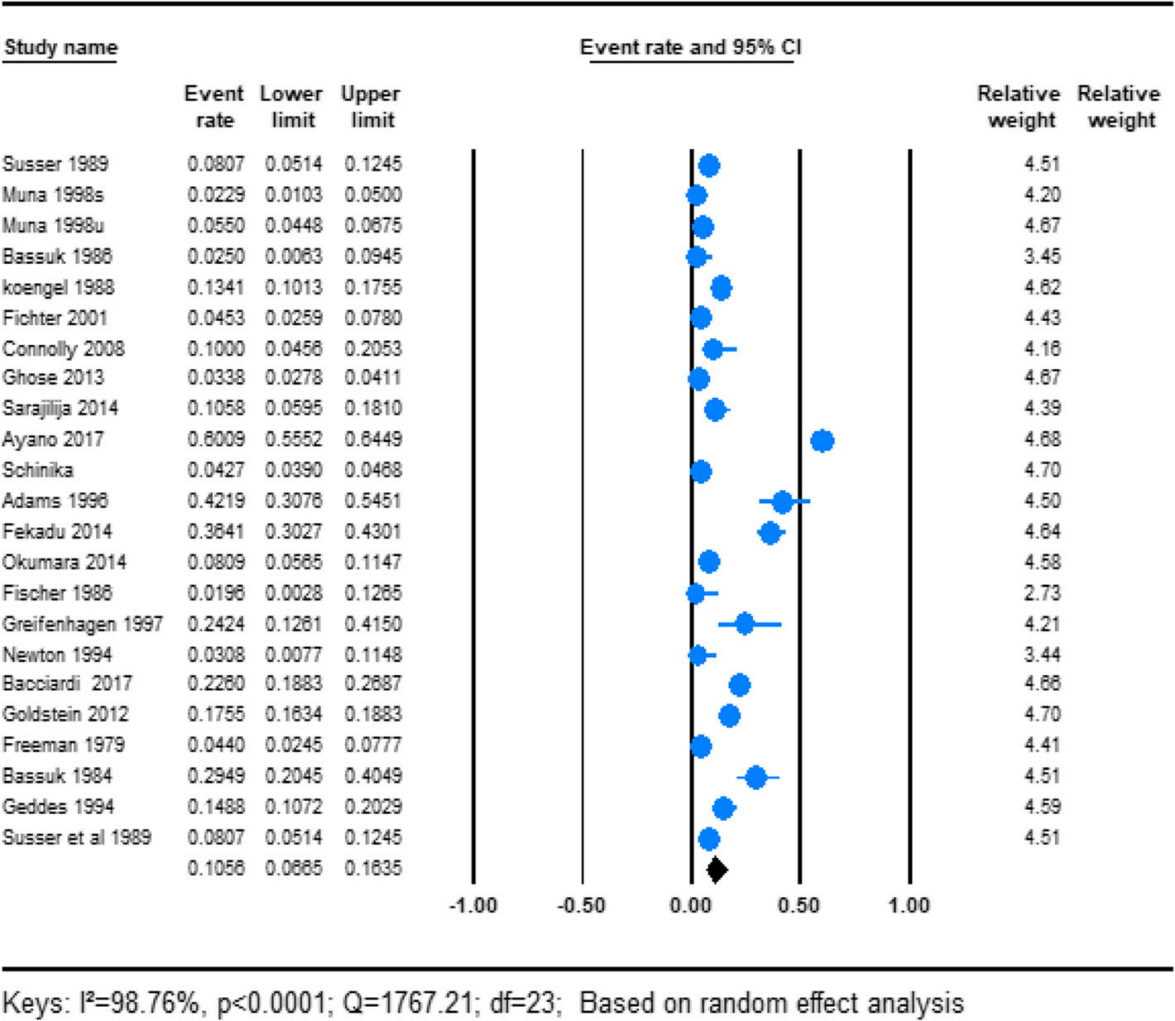
forest plot of the prevalence of schizophrenia among homeless people. This figure illustrates the results of the meta-analysis of the studies on schizophrenia on homeless people suing random effect model

In this review 24 of the studies reported the prevalence of schizophrenia among homeless people. Analysis of those studies which reported prevalence the prevalence of schizophrenia we found that the prevalence of schizophrenia was 10.29% (95%CI 6.44–16.02). Significant heterogeneity was found for this analysis (*I*^2^ = 98.76; *p* < 0.001) (Fig. 3).

#### The prevalence of the schizophreniform disorder in homeless people

**Fig. 4:**
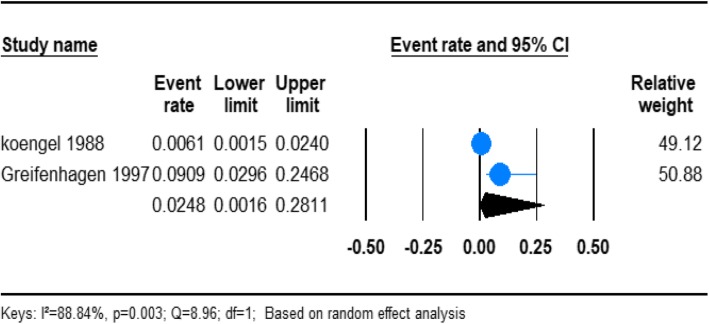
Forest plot of the prevalence of schizophreniform disorders among homeless people. The figure shows the results of the meta-analysis of the studies on schizophreniform disorders on homeless

From the total, 2 of the studies reported the prevalence of schizophreniform disorder in homeless people (Table 1). Based on the results of the random-effects method, the pooled prevalence estimates of schizophreniform disorder in the homeless people was 2.48% (95% CI; 0.16–28.11). We found significant heterogeneity for this analysis (*I*^2^ = 88.84%; *p* < 0.001) (Fig. 4).

#### The prevalence of the schizoaffective disorder in homeless people

**Fig. 5:**
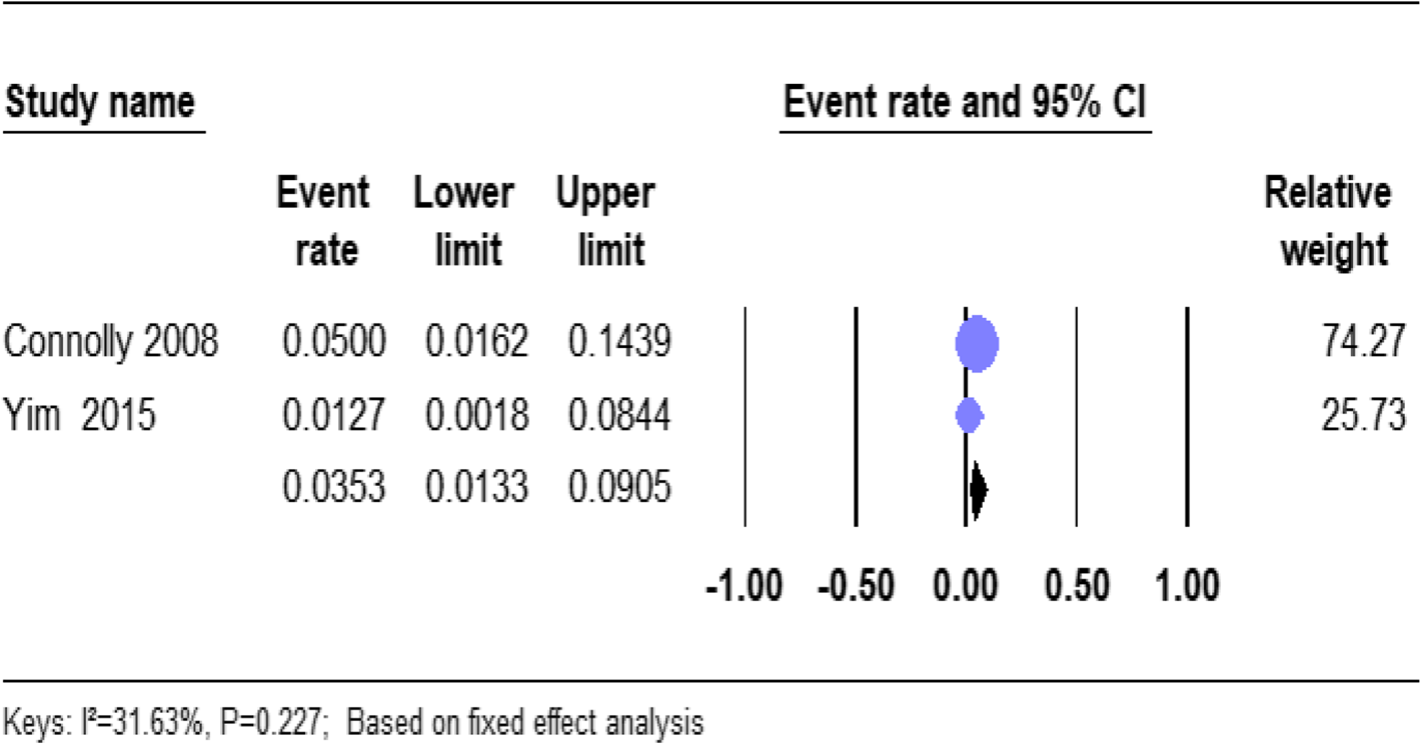
forest plot of the prevalence of schizoaffective disorders among homeless people. The figure shows the results of the meta-analysis of the studies on schizoaffective disorders on homeless

From the total, 2 of the studies reported the prevalence of schizoaffective disorder in homeless people (Table 1). Based on the results of the fixed-effects method, the pooled prevalence estimates of schizoaffective disorder in the homeless people was 3.53% (95% CI; 1,33–9.05). We found no significant heterogeneity for this analysis (*I*^2^ = 31.63%; *p* = 0.227). (Fig. 5).

#### The prevalence of psychotic disorder NOS in homeless people

**Fig. 6:**
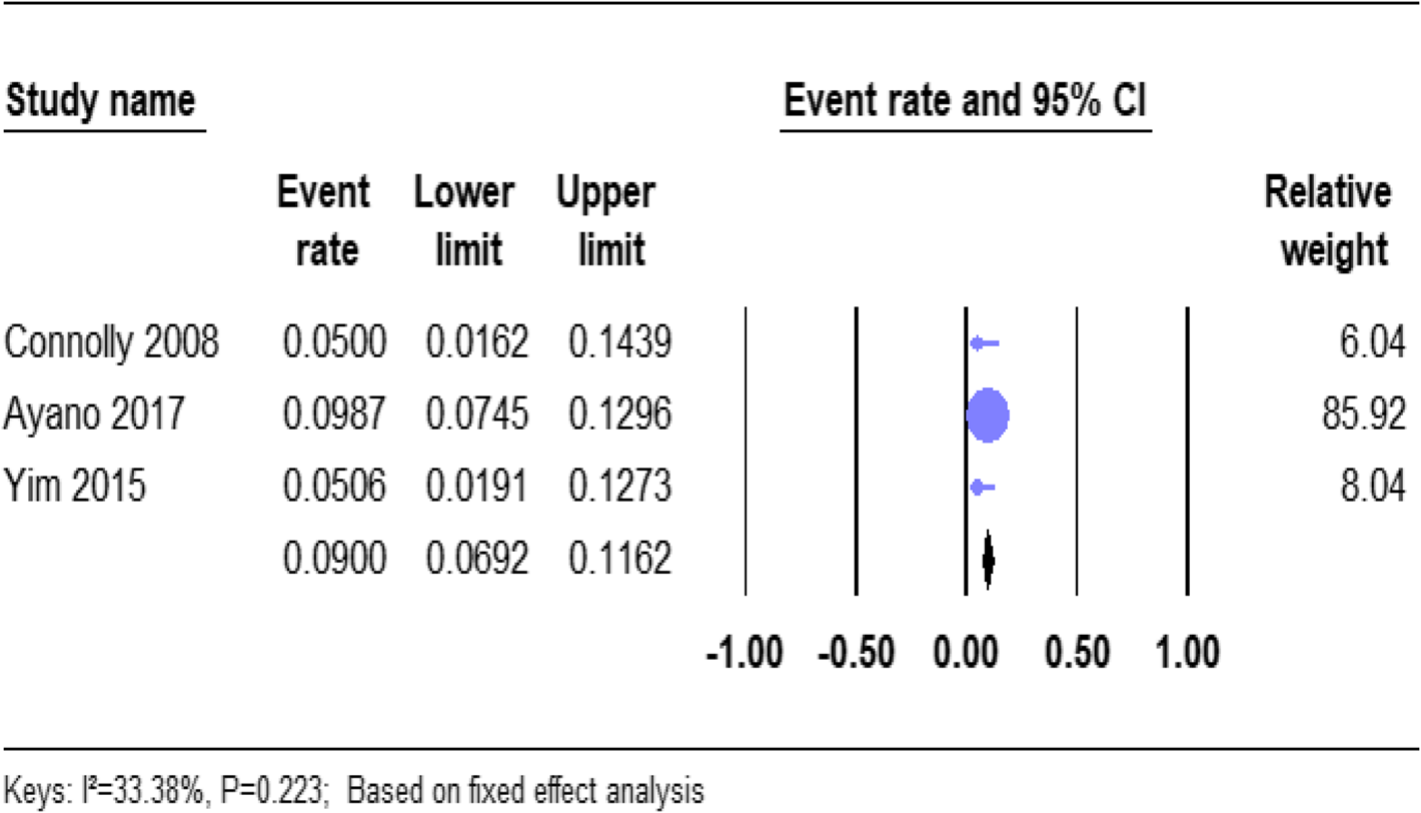
Forest plot of the prevalence of psychotic disorders NOS among homeless people. The figure shows the results of the meta-analysis of the studies on psychotic disorders not otherwise specified (NOS) on homeless

From the total, 3 of the studies reported the prevalence of psychotic disorder NOS in homeless people (Table 1). Based on the results of the fixed-effects method, the pooled prevalence estimates of psychotic disorder NOS in the homeless people was 9.00% (95% CI; 6.92–11.62). We found no significant heterogeneity for this analysis (*I*^2^ = 33.38%; *p* = 0.223) (See Fig. 6).

### Sensitivity analysis

To further explore the possible source of heterogeneity in the analysis of the prevalence of psychosis in the homeless people, we conducted a sensitivity analysis by the country of origin, the instrument used to measure psychotic disorders and the quality of the included studies. When limiting the analysis to the country origin we found that the prevalence of psychotic disorder was 18.80% for developed countries and it was 29.16% for developing countries. In these sensitivity analyses, the observed difference in the magnitude of overall psychotic disorder between the countries was not statistically significant (*P* = 0.313). Similarity to overall psychotic disorder we found no significant difference in the prevalence of schizophrenia across the countries (*P* < 0.001). The pooled prevalence estimate of schizophrenia was highest in developing countries (22.15%) as compared with developed countries (8.83%). In our sensitivity analysis based on the instrument used to measure psychotic disorder the pooled estimated prevalence of overall psychosis was 17.42% as measured by diagnostic instrument and it was 32.19% as measured by screening instrument, although the difference observed was not statistically significant (*P* = 0.256). The prevalence of schizophrenia was 13.20% (95%CI 6.05–26.44) for studies conducted in or after the year 2010 and it was 8.88% (95%CI 5.47–14.09) for studies conducted before 2010, whereas the prevalence of psychosis was 24.44% (95%CI 13.65–29.03) for studies conducted in or after 2010 and it was 20.09% (95%CI 10.18–28.49) for studies conducted before 2010. In fact, the observed difference in the prevalence of schizophrenia and psychosis by year of the study was not statically significant (*P* = 0.386 for schizophrenia and *P* = 0.392 for psychosis). Finally, we also conducted the sensitivity analysis based on the quality of included studies, the prevalence of overall psychotic disorder was found relatively equal for good quality (21.37%) and fair quality studies (22.67%), and the difference was not statistically significant (*P* = 0.868). (Table 2).

**Table 2:** Sensitivity analysis of all studies based on the country of origin, the instrument used, and study quality of the included studies

Subgroups	Studies, n	Prevalence (%)	95%CI	Disorder type	Heterogeneity across the studies		Heterogeneity between groups (*P-value*)
					I^2^	*P*-value	
Country							
Developed	13	18.80	11.90–28.41	Overall psychotic disorder	99.42	< 0.001	0.313
Developing	5	29.16	13.39–52.28		99.92	< 0.001	
Instrument used							
Screening	5	32.19	13.51–59.08	Overall psychotic disorder	98.97	< 0.001	0.256
Diagnostic	13	17.42	8.26–30.08		99.78	< 0.001	
Quality of studies							
High	9	21.37	11.50–36.24	Overall psychotic disorder	99.71	< 0.001	0.868
Fair	9	22.60	16.22–30.57		79.34	< 0.001	
Country							
Developed	19	8.83	4.28–13.64	Schizophrenia	98.06	< 0.001	0.080
Developing	4	22.15	1.83–43.98		97.22	< 0.001	
Year							
Before 2010	14	8.88	5.47–14.09	Schizophrenia	92.59	< 0.001	0.386
2010 and above	9	13.20	6.05–26.44		99.49	< 0.001	
Year							
Before 2010	8	20.09	10.18–28.49	Psychosis	93.92	< 0.001	0.392
2010 and above	10	24.44	13.65–29.03		99.68	< 0.001	

### Meta-regression

We first conducted an initial univariate regression analysis, which guided the selection of the independent variables to include in the final meta-regression model. As recommend all variables with *P*-value < 0.8 were included in the final regression analysis [64]. The impacts of year (studies conducted before 2010 and 2010 and above), country status (developing and developed), and, and instrument used to measure the disorders (screening and diagnostic) were quantified in the meta-regression model. The analysis was conducted for both schizophrenia and psychosis. The overall proportion of variance explained by the above covariates in the final model was 23% for schizophrenia (*R*^2^ = 23%; *P* value = 0.5880) and its was 0.0% for psychosis (*R*^2^ = 0.0%; *P* value = 0.4415). All three covariates such as year, country status and diagnostic instruments were not statistically significant determinants for the observed variation in the prevalence across studies for both psychosis and schizophrenia. (Additional files 4 and 5).

### Publication bias

**Fig. 7:**
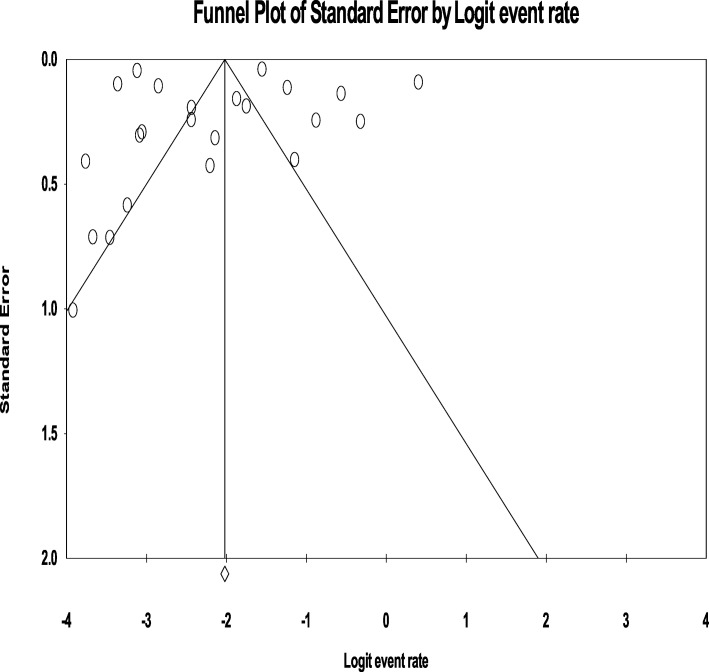
Publication bias for schizophrenia among homeless people

**Fig. 8:**
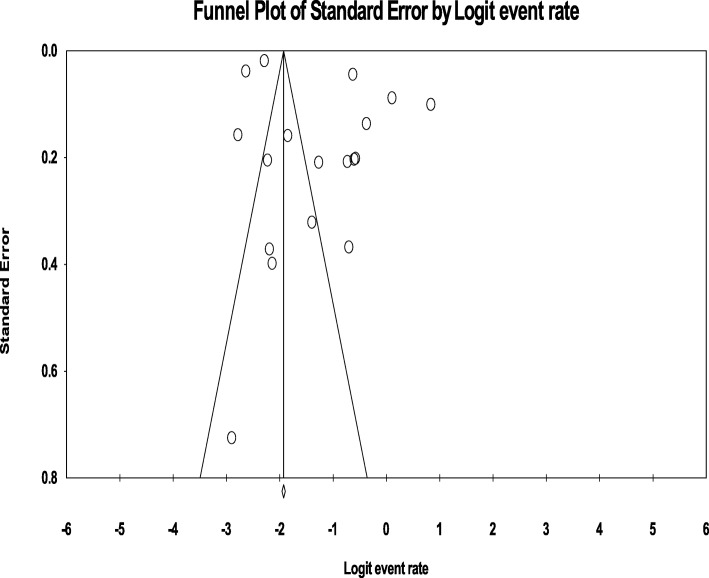
Publication bias for psychosis among homeless people

The funnel plot was symmetric and Egger’s regression tests provided no evidence of substantial publication bias for the prevalence of overall psychotic disorder (B = 6.94, SE = 3.83, *P* = 0.089) as well as schizophrenia (B = 0.276, SE = 2.84, *P* = 0.923). (Fig. 7 and 8).

## Discussion

To the best of our knowledge, this is so far the first study to systematically search, assess, and synthesize the estimated poled prevalence of schizophrenia and other psychotic disorders among homeless people. We conducted a systematic review and meta-analysis over 31 studies on the prevalence of schizophrenia and other psychotic disorders among homeless people. Our analysis resulted in a remarkably higher prevalence of overall psychosis (21.21%), schizophrenia (10.29%), schizophreniform disorder (2.48%), schizoaffective disorder (3.53%), as well as psychotic disorders not otherwise specified (9%) among homeless people The findings of our meta-analysis highlighted that the prevalence of schizophrenia and other psychotic disorders were higher among homeless people as compared to the reported prevalence in the general population. These findings underline schizophrenia and other psychotic disorders as a significant and serious global public health issue among homeless people.

The present systematic review and meta-analysis revealed that the existing epidemiologic data on the prevalence of schizophrenia and other psychotic among homeless people demonstrated a considerable difference based on the country (the origin of the study), the tools used to quantify schizophrenia and other psychotic disorders, and the reported quality of the studies. The majority of the included articles were performed in developed countries (87.1%; *n* = 27) and only a small number of studies were conducted in developing countries (12.90%; *n* = 4). To quantify the magnitude of schizophrenia and other psychotic among the homeless, some of the articles used screening tools and some used diagnostic instruments.

The results of our study underlined that the prevalence estimates of overall psychosis (21.21%) were remarkably higher than the previous systematic review reports from western countries among the homeless (12.7%) [65]. The possible reasons for the observed difference includes: first, the previous review considered specific categories of psychotic disorders including schizophrenia, schizoaffective and other distinct categories of psychotic disorders as psychosis which obviously underestimates the estimated prevalence whereas in the current meta-analysis, studies that reported the estimate of overall psychosis only were included under psychosis analysis and for articles that reported the specific categories of psychotic disorder we conducted separate analysis; second, the present review included studies from developed as well as developing countries whereas the previous study was conducted only in western countries and in our subgroup analysis we found that the magnitude of psychosis in developed countries was lower than that of developing countries. Therefore, the possible reason for the higher magnitude of psychosis in the previous study may be due to the inclusion of new studies including studies from developing countries.

The pooled prevalence estimates of schizophrenia in the general population have been found to be (0.4%) [66], and the pooled prevalence estimates in the current study exceed this estimate more than 25 fold (10.29%). The current systematic review and meta-analysis also resulted in a remarkably greater prevalence of schizophreniform disorder (2.48%), schizoaffective disorder (3.53%), and any psychosis (21.21%) among the homeless. These results are higher than the reported prevalence in the general population [67]. There are a range of explanations for the observed remarkable difference in the magnitude of schizophrenia between homeless people and general populations: First, the presence of a considerably higher prevalence rates of serious physical health problems such as tuberculosis, HIV/AIDS and other medical conditions possibly could contribute to the greater prevalence of psychiatric disorders including schizophrenia among the homeless people [23, 68–72]; second, evidence shows that homeless people are more likely to experience traumatic events including physical and sexual abuse, which has been linked with greater risks of mental health problems including psychotic disorders in those who are exposed [73–76]; thirdly, the presence of considerably higher magnitude of mental, neurologic and substance use disorders among homeless people including anxiety, depressive, bipolar, and other psychiatric and substance use disorder among homeless people as compared with other populations are the other possible reasons for the observed high prevalence of schizophrenia among the homeless [7, 8, 40, 77].

This review revealed the highest prevalence of schizophrenia and other psychotic disorders was higher in developing countries than in developed countries. There are many interpretations of the observed variation in the magnitude of schizophrenia and other psychotic disorders among homeless people among the countries. (1), the possible methodologic variations such as the tools utilized to quantify schizophrenia and other psychotic disorders among the included studies is the possible reason for the observed considerable difference. For instance, in our subgroup analysis according to the tools used we found we found a remarkably high prevalence of psychosis as measured by screening instrument (32.19%) than diagnostic instruments (17.42%); (2) the possible variations in in the magnitude of the potential contributing factors that increase the risk of schizophrenia and other psychotic disorders among the study participants across the countries including potential life treating traumatic events and disasters and serious physical health problems are the other possible explanations for the observed variation in the magnitude of schizophrenia and other psychotic disorders across the nations; (3) the variations in the severity of the disease and comorbid other psychiatric and substance-related disorders among homeless people across the countries are the other potential reasons for the observed difference. In fact, the observed the difference in magnitude of schizophrenia psychosis among the developed and developing countries was not statically significant. Therefore, the difference is a chance of finding rather than a real difference. Additionally, the lower number of studies in developing countries as compared with developed countries must be considered. Finally, the inclusion of studies conducted on-street homeless in developing countries than developed might overestimated the prevalence.

### Strength and limitations

The present systematic review and meta-analysis had numerous strengths: First, the study utilized predefined procedure for searching selection of the articles, data extraction as well as analysis; second, the extradition of information from the included articles and quality evaluation were performed by two independent; third, we conducted a subgroup and sensitivity analysis based on the country of the study, the tools used, and the quality of the studies. Finally, we also conducted a meta-regression analysis to quantify the impacts of important covariates such as year of study, the status of the country and instruments used to quantify psychotic disorders. The meta-regression provided no evidence of a significant effect by all of the above covariates.

However, this review also had some limitation: first, most of the included studies were conducted in developed countries and only 4 studies were conducted in developing countries; second, we included small number of articles a subgroup and sensitivity analysis which could possibly reduce the power of the estimate; third, we observed a considerable heterogeneity across the studies.

### The implication of the findings for future research and clinical practice

This study has found some implication for the future clinical practice as well as research; Firstly, we found a higher magnitude of schizophrenia and other psychotic disorders among homeless people as compared to the reported magnitude in the general population, which needs further strong studies to explore the possible reasons for the higher magnitude. Secondly, we found a few numbers of studies that estimated the prevalence of schizophrenia and other psychotic disorders in developed countries. So, further studies are required to understand the prevalence of schizophrenia and other psychotic disorders in developing countries and compare them with the prevalence estimate from developed countries. Thirdly, attention need to give by the concerned bodies to address schizophrenia and other psychotic disorders among homeless people including better ways of prevention and treatment strategies possibly integrating the mental health service of the homeless with the existing medical services, which suggests utilizing holistic approaches to address the health needs of the homeless people such as physical, mental, and substance use problems as well as suicide.

## Conclusion

In conclusion, the current systematic review and meta-analysis revealed that the prevalence of schizophrenia and other psychotic disorders are notably high among homeless people. The meta-analysis also showed a higher prevalence of both psychosis and schizophrenia in developing countries as compared to developed countries. Robust studies determining the possible reasons for the higher magnitude of schizophrenia and other psychotic disorders among the homeless were warranted. Moreover, there is an urgent need for robust and informative studies to help develop better mechanisms of prevention, detection as well as treatment of those disorders among homeless people.

## Supplementary information

**Additional file 1. **Screenshot of document results from the three databases. This additional material shows snapshot of number of studies identified during the database search PubMed (*n* = 2511), Embase (*n* = 659), and Scopus (*n* = 247).

**Additional file 2.** Excluded full text articles with reasons of exclusion. The reasons for the excluded full text articles includes: (1) not reported prevalence; (2) reviews; (3) duplicate; (4) not conducted on homeless people.

**Additional file 3.** The Quality of the included studies based on the NOS quality score, a 9 point score. Score 7 and above represented good quality; 2 to 6 represented fair quality; and 0 and 1 represented poor quality.

**Additional file 4.** Meta-regression of the prevalence of psychosis among the homeless based on year, tools used and country status.

**Additional file 5.** Meta-regression of the prevalence of schizophrenia among the homeless based on year, tools used and country status.

## Data Availability

All data generated or analyzed during this study are included in this article.

## Abbreviations

AIDS: Acquired Immune Deficiency Syndrome
CANSAS: Camberwell Assessment of Need Short Appraisal Schedule
CIDI: Composite International Neuropsychiatric Interview
DSM IV-TR: Diagnostic Statistical Manual of Mental disorders fourth, Edition, text revised
DSM-5-TR: Diagnostic Statistical Manual of Mental disorders fifth, Edition, text revised
HIV: Human Immune Deficiency Virus
ICD: International Classification of Disease
MINI: Mini International Neuropsychiatric Interview
NOS: Newcastle-Ottawa Scale
PRISMA: Preferred Reporting Items for Systematic Reviews and Meta-Analyses
PSE: Present State Examination
UK: United Kingdom
USA: United States of America
WHO: World Health Organization
