# Supplementary figures and images for: The prevalence of schizophrenia and other psychotic disorders among homeless people: a systematic review and meta-analysis
Source: BMC Psychiatry. 2019 Nov 27;19:370. doi: 10.1186/s12888-019-2361-7 (PMC6880407; doi:10.1186/s12888-019-2361-7)

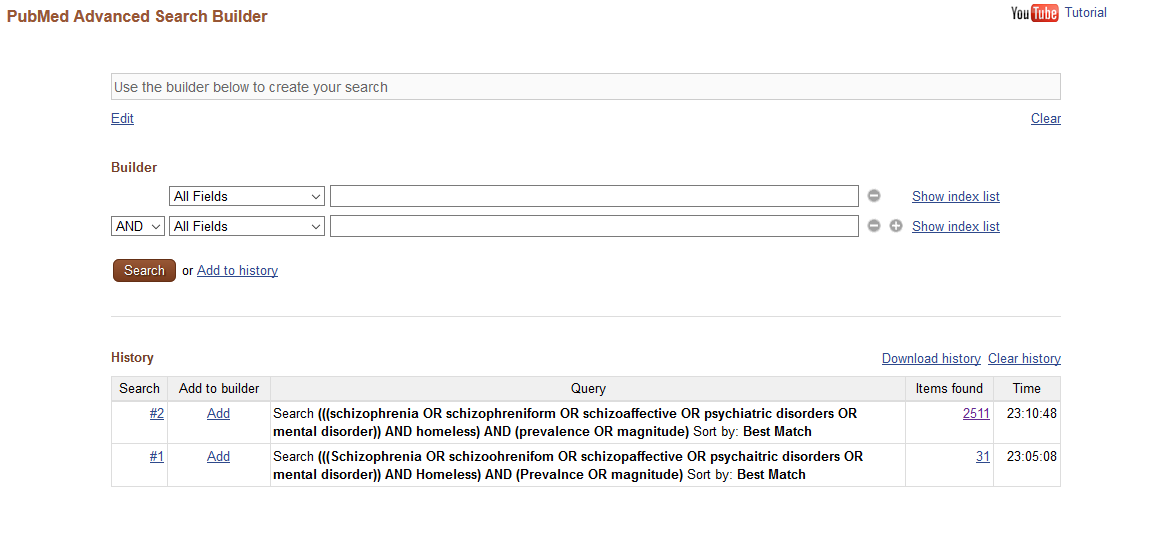


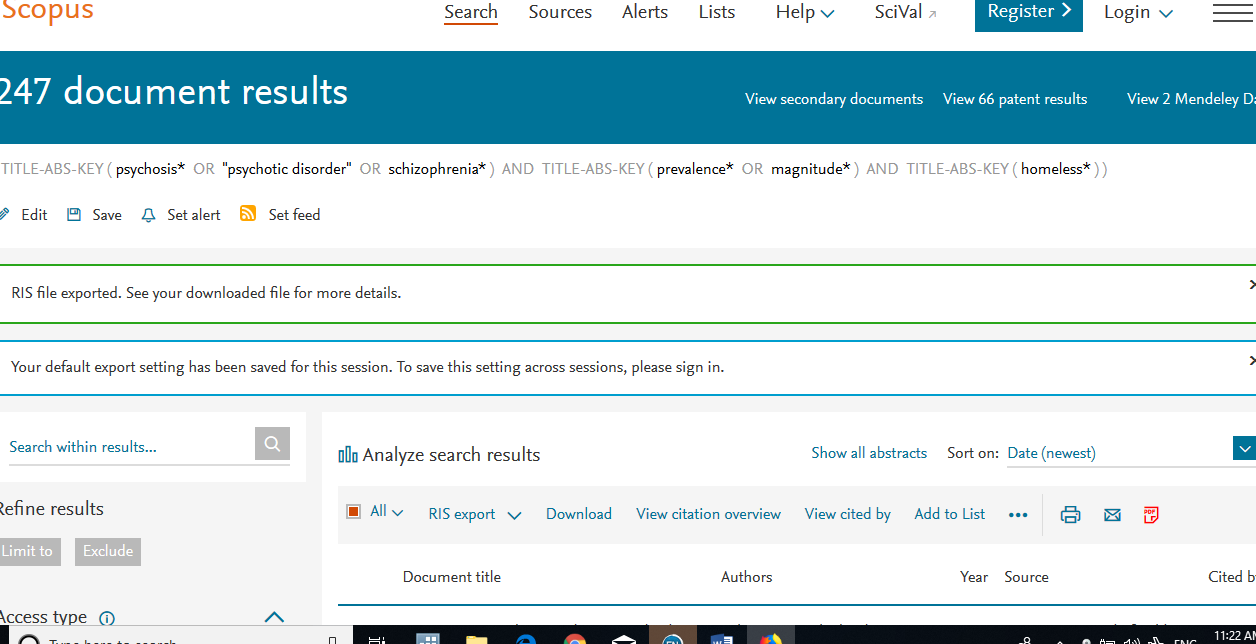


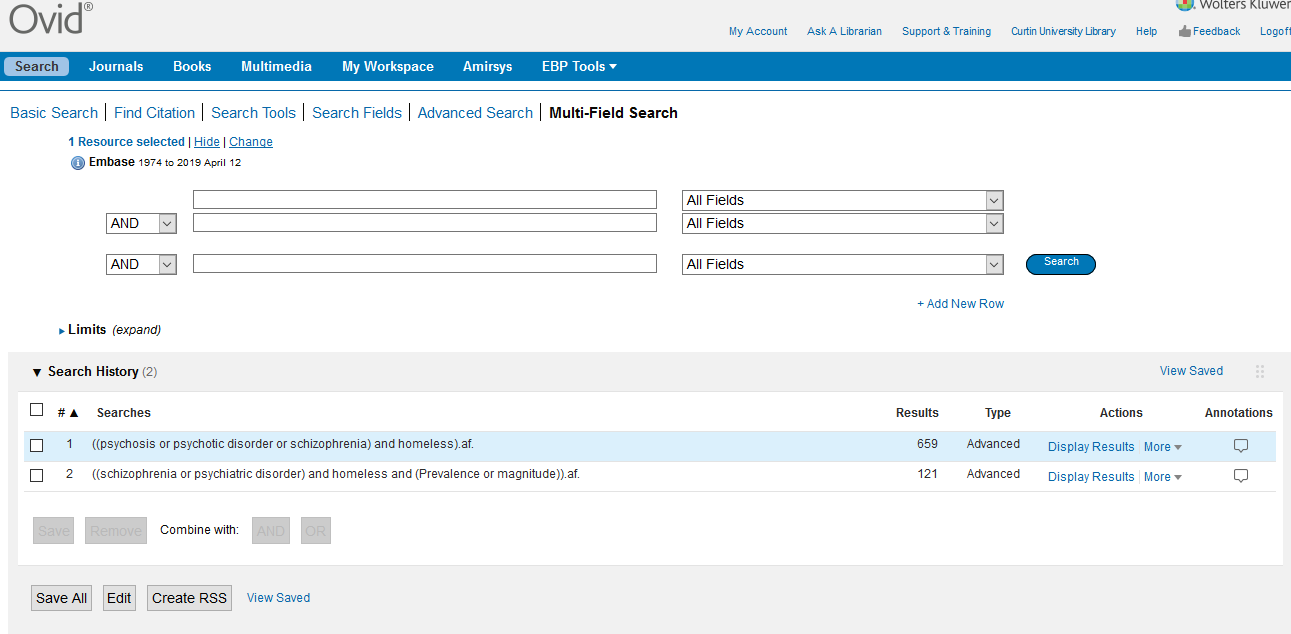

Supplement: Supplementary file 1 — Additional file 1. Screenshot of document results from the three databases. This additional material shows snapshot of number of studies identified during the database search PubMed (n = 2511), Embase (n = 659), and Scopus (n = 247). [file 12888_2019_2361_MOESM1_ESM.docx]
