# Supplementary material for: The prevalence of schizophrenia and other psychotic disorders among homeless people: a systematic review and meta-analysis
Source: BMC Psychiatry. 2019 Nov 27;19:370. doi: 10.1186/s12888-019-2361-7 (PMC6880407; doi:10.1186/s12888-019-2361-7)
Supplement: Supplementary file 2 — Additional file 2. Excluded full text articles with reasons of exclusion. The reasons for the excluded full text articles includes: (1) not reported prevalence; (2) reviews; (3) duplicate; (4) not conducted on homeless people. [file 12888_2019_2361_MOESM2_ESM.docx]

**Supplementary file 3:** Excluded full text articles with reasons of exclusion

The reasons for the excluded full text articles includes: (1) Not reported prevalence (1-9); (2) reviews (10); (3) duplicate (11-13); (4) not conducted on homeless people (14-19).

1. Van Straaten B, Schrijvers CT, Van der Laan J, Boersma SN, Rodenburg G, Wolf JR, et al. Intellectual disability among Dutch homeless people: prevalence and related psychosocial problems. PloS one. 2014;9(1):e86112.

2. O'Brien KK, Schuttke A, Alhakeem A, Donnelly-Swift E, Keogh C, O'Carroll A, et al. Health, perceived quality of life and health services use among homeless illicit drug users. Drug and alcohol dependence. 2015;154:139-45.

3. Bellavia CW, Toro PA. Mental disorder among homeless and poor people: a comparison of assessment methods. Community mental health journal. 1999;35(1):57-67.

4. Adams CE, Pantelis C, Duke PJ, Barnes TR. Psychopathology, social and cognitive functioning in a hostel for homeless women. The British journal of psychiatry : the journal of mental science. 1996;168(1):82-6.

5. Bassuk EL, Rubin L, Lauriat AS. Characteristics of sheltered homeless families. American journal of public health. 1986;76(9):1097-101.

6. Gelberg L, Linn LS. Psychological distress among homeless adults. The Journal of nervous and mental disease. 1989;177(5):291-5.

7. Greenberg GA, Rosenheck RA. Jail incarceration, homelessness, and mental health: a national study. Psychiatric services (Washington, DC). 2008;59(2):170-7.

8. Haugland G, Siegel C, Hopper K, Alexander MJ. Mental illness among homeless individuals in a suburban county. Psychiatric services (Washington, DC). 1997;48(4):504-9.

9. Andersen P, Knop J, Almdal T, Odum N, Schulsinger F. [Mental and social problems of homeless men. A study of the shelter population in Copenhagen]. Ugeskrift for laeger. 1984;146(14):1074-9.

10. Fazel S, Khosla V, Doll H, Geddes J. The prevalence of mental disorders among the homeless in western countries: systematic review and meta-regression analysis. PLoS medicine. 2008;5(12):e225.

11. Fichter M, Quadflieg N. Alcoholism in homeless men in the mid-nineties: results from the Bavarian Public Health Study on homelessness. European archives of psychiatry and clinical neuroscience. 1999;249(1):34-44.

12. Kovess V, Lazarus CM. The prevalence of psychiatric disorders and use of care by homeless people in Paris. Social psychiatry and psychiatric epidemiology. 1999;34(11):580-7.

13. Fletcher JB, Reback CJ. Mental health disorders among homeless, substance-dependent men who have sex with men. Drug and alcohol review. 2017;36(4):555-9.

14. Goodhew M, Salmon AM, Marel C, Mills KL, Jauncey M. Mental health among clients of the Sydney Medically Supervised Injecting Centre (MSIC). Harm reduction journal. 2016;13(1):29.

15. Greacen T, Finkelstein C. Mental health care in Paris. European psychiatry : the journal of the Association of European Psychiatrists. 2005;20 Suppl 2:S285-8.

16. Hamdullahpur K, Jacobs KWJ, Gill KJ. A comparison of socioeconomic status and mental health among inner-city Aboriginal and non-Aboriginal women. International journal of circumpolar health. 2017;76(1):1340693.

17. Kar SK, Sharma E, Agarwal V, Singh SK, Dalal PK, Singh A, et al. Prevalence and pattern of mental illnesses in Uttar Pradesh, India: Findings from the National Mental Health Survey 2015-16. Asian journal of psychiatry. 2018;38:45-52.

18. Kisely S, Chisholm P. Shared mental health care for a marginalized community in inner-city Canada. Australasian psychiatry : bulletin of Royal Australian and New Zealand College of Psychiatrists. 2009;17(2):130-3.

19. Albert M, McCaig LF. Emergency Department Visits Related to Schizophrenia Among Adults Aged 18-64: United States, 2009-2011. NCHS data brief. 2015(215):1-8.
