# Supplementary material for: The prevalence of schizophrenia and other psychotic disorders among homeless people: a systematic review and meta-analysis
Source: BMC Psychiatry. 2019 Nov 27;19:370. doi: 10.1186/s12888-019-2361-7 (PMC6880407; doi:10.1186/s12888-019-2361-7)
Supplement: Supplementary file 3 — Additional file 3. The Quality of the included studies based on the NOS quality score, a 9 point score. Score 7 and above represented good quality; 2 to 6 represented fair quality; and 0 and 1 represented poor quality. [file 12888_2019_2361_MOESM3_ESM.docx]

**Table 1:** The Quality of the included studies based on the NOS quality score, a 9 point score

| Author(year) (reference number) | Nos score | Quality |
| --- | --- | --- |
| ([Susser et al., 1989](#_ENREF_56)) | 8 | Good |
| ([Munoz et al., 1998](#_ENREF_38)) | 9 | Good |
| ([BASSUK et al., 1986](#_ENREF_4)) | 5 | Fair |
| ([Koegel et al., 1988](#_ENREF_32)) | 8 | Good |
| ([Fichter and Quadflieg, 2001](#_ENREF_15)) | 8 | Good |
| ([Längle et al., 2005](#_ENREF_34)) | 6 | Fair |
| ([Connolly et al., 2008](#_ENREF_8)) | 5 | Fair |
| ([Ghose et al., 2013](#_ENREF_25)) | 9 | Good |
| ([Tsai et al., 2014](#_ENREF_59)) | 9 | Good |
| ([Noe et al., 2016](#_ENREF_43)) | 9 | Good |
| ([Sarajlija et al., 2014](#_ENREF_51)) | 6 | Fair |
| ([Ayano et al., 2017](#_ENREF_1)) | 8 | Good |
| ([Schinka et al., 2012](#_ENREF_52)) | 9 | Good |
| ([Topolovec-Vranic et al., 2017](#_ENREF_58)) | 9 | Good |
| ([Fletcher and Reback, 2017](#_ENREF_17)) | 8 | Good |
| ([Cuvee.Adams et al., 1996](#_ENREF_9)) | 5 | Fair |
| ) ([Fekadu1 et al., 2014](#_ENREF_14)) | 8 | Good |
| ([Okamura et al., 2014](#_ENREF_44)) | 8 | Good |
| ([Prinsloo et al., 2012](#_ENREF_47)) | 5 | Fair |
| ([Kovess and Lazarus, 1999](#_ENREF_33)) | 9 | Good |
| ([Larney et al., 2009](#_ENREF_36)) | 5 | Fair |
| ([FISCHER et al., 1986](#_ENREF_16)) | 5 | fair |
| (Greifenhagen et.al.  1997) | 4 | Fair |
| ([NEWTON et al., 9194](#_ENREF_39)) | 5 | Fair |
| ([BACCIARDI et al., 2017](#_ENREF_2)) | 8 | Good |
| ([Goldstein et al., 2012](#_ENREF_26)) | 9 | Good |
| ([Freeman et al., 1979](#_ENREF_21)) | 8 | Good |
| ([Bassuk et al., 1984](#_ENREF_3)) | 5 | Fair |
| ([Geddes et al., 1994](#_ENREF_23)) | 8 | Good |
| Cougnard et.al. 2006 | 6 | Fair |
| Yim et.al 2015 | 5 | Fair |
| **Key:** Score 7 and above represented good quality; 2 to 6 represented fair quality; and 0 and 1 represented poor quality | | |
